# The value of peripheral blood PLR, Lp-PLA2, MHR, SII, and HCY in assessing the rupture risk of small and medium-sized intracranial aneurysms

**DOI:** 10.3389/fneur.2026.1729462

**Published:** 2026-02-17

**Authors:** Chaojun Yan, Xinyu Lu

**Affiliations:** Department of Neurosurgery, People’s Hospital Affiliated to Jiangsu University, School of Medicine Jiangsu University, Zhenjiang, China

**Keywords:** aneurysm rupture risk, inflammatory metabolism, peripheral blood biomarkers, predictive model, small and medium-sized intracranial aneurysms

## Abstract

**Objective:**

We aimed to investigate the relationship of peripheral blood platelet-to-lymphocyte ratio (PLR), lipoprotein-associated phospholipase A2 (Lp-PLA2), monocyte-to-high-density lipoprotein ratio (MHR), systemic immune-inflammation index (SII), and Homocysteine (HCY) with risk of rupture for small to medium-sized intracranial aneurysms, and examine their combined value as potential predictive markers.

**Methods:**

We conducted a retrospective analysis of clinical data from 80 patients with intracranial aneurysms who underwent endovascular embolization from January 2022 to January 2025. Patients were divided into a ruptured group (*n* = 27) and an unruptured group (*n* = 53). Associations between biomarkers and rupture status were evaluated using univariate and multivariate logistic regression. A predictive nomogram was constructed and assessed using calibration, receiver operating characteristic (ROC) curve analysis, decision curve analysis (DCA), and bootstrap internal validation.

**Results:**

Levels of PLR, Lp-PLA2, MHR, SII, and HCY were significantly higher in ruptured cases, while SOD and IL-10 were significantly lower (*p* < 0.05). In multivariable logistic regression, all five biomarkers were associated with rupture. The combined biomarker model showed high apparent discrimination (AUC = 0.969) and was internally validated using bootstrap resampling; however, given the small, single-center sample and the lack of adjustment for key clinical and morphological predictors, the model requires cautious interpretation and independent validation.

**Conclusion:**

PLR, Lp-PLA2, MHR, SII, and HCY were associated with rupture status in small to medium-sized intracranial aneurysms. A combined multi-biomarker nomogram showed strong apparent discrimination; however, the incremental value beyond established clinical and morphological predictors and the generalizability of this model need confirmation in larger, preferably multicenter cohorts.

## 1 Introduction

Intracranial aneurysm (IA) is a prevalent form of cerebrovascular disease, and rupture can result in debilitating complications such as subarachnoid hemorrhage (SAH) associated with dire rates of mortality and morbidity (1). Currently, clinical management of unruptured intracranial aneurysms (UIAs) is primarily based on morphological features (e.g., size and neck width) and previously reported scoring systems (e.g., PHASES and ELAPSS) (2–4). These methodologies illustrate certain aspects of the potential underlying biological mechanisms influencing aneurysm instability but do not include dynamic inflammatory or metabolic biomarkers, which are increasingly recognized as mediating factors influencing rupture risk.

Cerebrovascular research has increasingly highlighted the contributions of chronic inflammation, endothelial dysfunction, oxidative stress, and metabolic dysregulation to the biochemistry and pathogenesis of intracranial aneurysms. In particular, sustained inflammatory activity, coupled with degradation of the extracellular matrix, may further destabilize the aneurysm wall and compromise its structural integrity—processes in which loss of vascular smooth muscle cell viability (e.g., via apoptosis) plays a critical role (5–7). Such pathophysiological changes are often reflected in circulating biomarkers. For instance, the platelet-to-lymphocyte ratio (PLR) serves as a well-established indicator of systemic inflammation and vascular disease progression. Lipoprotein-associated phospholipase A2 (Lp-PLA2), known to promote atherosclerotic plaque instability, may similarly undermine the structural strength of the aneurysm wall (8, 9). The monocyte-to-high-density lipoprotein ratio (MHR) and systemic immune-inflammation index (SII) integrate inflammatory and lipid-metabolic signals and have been associated with vascular lesion activity (10, 11). Moreover, homocysteine (HCY), a sulfur-containing amino acid, exacerbates oxidative stress and endothelial dysfunction, potentially accelerating aneurysm degeneration (12).

Notably, recent studies have proposed multi-biomarker models for risk stratification in cerebrovascular diseases (13, 14). These advances underscore the importance of complementing traditional morphological assessments with inflammatory and metabolic biomarkers. Nevertheless, the synergistic potential of a composite biomarker panel—and its clinical utility in predicting rupture risk specifically for small to moderate-sized aneurysms (≤15 mm)—remains largely unexplored.

To address this gap, we performed a retrospective analysis of patients with small and medium-sized intracranial aneurysms, examining the associations of PLR, Lp-PLA2, MHR, SII, and HCY with rupture status. We assessed the diagnostic and predictive performance of these biomarkers individually and in combination. By developing a multifactorial predictive model, this study aims to improve early identification of individuals at high risk of aneurysmal subarachnoid hemorrhage or rupture, thereby facilitating more personalized risk-management strategies.

## 2 Methods

### 2.1 Patients

In this retrospective study, we evaluated patients diagnosed with IA admitted to the Affiliated Hospital of Jiangsu University from January 2022 to January 2025. Eighty patients were included in the derivation cohort used for model development. In addition, an independent validation cohort of 50 patients (17 ruptured, 33 unruptured) treated at the same center between March 2022 and December 2024, meeting identical inclusion and exclusion criteria and not included in the derivation cohort, was used to test model performance in an independent sample.

Patients were categorized as unruptured (UA, *n* = 53) or ruptured (RA, *n* = 27) according to admission status. Inclusion criteria were: (1) IA confirmed by digital subtraction angiography (DSA) or high-resolution MRI with maximum aneurysm diameter ≤15 mm; (2) age ≥18 years; (3) treated with coil embolization with or without stent assistance; and (4) for RA, Hunt-Hess grade I-III at admission. Exclusion criteria were: cerebral arteriovenous malformation; intracranial tumor; severe hepatic or renal insufficiency; autoimmune disease; active bleeding tendency (INR > 1.5 or platelet 50 × 10^9^/L) or other coagulopathy; pregnancy or lactation; previous aneurysm surgery; or current anticoagulant therapy. Cases with technical failure of endovascular therapy (technical or infectious reasons) were excluded because these failures were procedure-related and not reflective of baseline rupture status, thereby reducing bias in biomarker analyses.

Because this was a retrospective cohort, several established clinical and radiological rupture predictors were incompletely captured in the medical record and imaging archive. In particular, detailed aneurysm morphology (e.g., aspect ratio and quantitative irregularity/daughter sac features), precise anatomical location subcategories, and comprehensive vascular risk factor and medication data (e.g., diabetes, lipid profile, statin/antiplatelet use, blood pressure measurements) were not consistently available for all patients. Basic aneurysm characteristics (maximum diameter, broad location category, and multilobulated shape) were available for most patients and are summarized in Table 1; however, a complete set of traditional predictors was only available in a subset, which limited inclusion of these variables in the primary multivariable models. Accordingly, the reported biomarker associations reflect adjustment for the included biomarkers and available clinical covariates, and residual confounding by unmeasured factors cannot be excluded.

**Table 1:** Baseline demographic, clinical, and morphological characteristics of patients with ruptured and unruptured aneurysms.

Variable	Unruptured (*n* = 53)	Ruptured (*n* = 27)	*p* value
Cases	53	27	
Sex: Male	31 (58.49%)	15 (55.56%)	0.802
Sex: Female	22 (41.51%)	12 (44.44%)	0.802
Age (years)	64.35 ± 8.97	65.34 ± 9.13	0.644
BMI (kg/m^2^)	23.46 ± 2.15	23.69 ± 2.04	0.647
Smoking	24 (45.28%)	10 (37.04%)	0.481
Drinking	27 (50.94%)	12 (44.44%)	0.582
Hypertension history	31 (58.49%)	21 (77.78%)	0.048
Max aneurysm diameter (mm)	5.4 (4.2–7.1)	7.9 (6.4–9.5)	0.002
Location: anterior circulation	41 (77.36%)	14 (51.85%)	0.017
Multilobulated shape	12 (22.64%)	11 (40.74%)	0.041

Continuous variables are presented as mean ± SD or median (IQR); categorical variables as count (%).

### 2.2 Biomarker assessment

Fasting venous blood was collected preoperatively and 1 week after the procedure. Samples were immediately anticoagulated and subsequently centrifuged to isolate plasma and serum for biomarker analysis. The platelet-to-lymphocyte ratio (PLR) was calculated as the platelet count divided by the lymphocyte count. Similarly, the monocyte-to-high-density lipoprotein ratio (MHR) was derived as monocyte count divided by high-density lipoprotein cholesterol (HDL-C) level. The systemic immune-inflammation index (SII) was computed as (platelet count × neutrophil count) / lymphocyte count (13, 14). Serum concentrations of lipoprotein-associated phospholipase A2 (Lp-PLA2), homocysteine (HCY), interleukin-6 (IL-6), tumor necrosis factor-α (TNF-α), interleukin-10 (IL-10), malondialdehyde (MDA), and superoxide dismutase (SOD) were quantified according to standardized laboratory protocols; detailed methods are provided in Supplementary Table S1.

### 2.3 Statistical analysis

Normality of continuous variables was assessed using the Shapiro–Wilk test. Continuous variables are presented as mean ± SD or median (IQR) and compared between groups using the independent-samples *t* test or Mann–Whitney U test, as appropriate; categorical variables were compared using the χ^2^ test or Fisher’s exact test. For within-patient pre−/post-treatment comparisons, paired-samples *t* tests or Wilcoxon signed-rank tests were used. Associations between biomarkers and rupture status (binary outcome) were evaluated using univariate and multivariable logistic regression; MHR was scaled per 0.1-unit increase to improve interpretability and numerical stability. Multicollinearity was assessed using variance inflation factors (VIF < 5 considered acceptable), and influence diagnostics (standardized residuals and Cook’s distance) were examined. Discrimination was evaluated using ROC curves and AUCs with 95% CIs (DeLong method), and AUCs between models were compared using the DeLong test. Calibration was assessed using a calibration curve and the Hosmer–Lemeshow goodness-of-fit test. Internal validation was performed using bootstrap resampling (1,000 iterations) to estimate optimism-corrected discrimination and calibration. Clinical net benefit was assessed using decision curve analysis (DCA). Spearman’s rank correlation was used only to describe correlations among continuous biomarkers. Complete-case analysis was applied; no imputation was performed.

The main multivariable analyses adjusted for available clinical factors (age, sex, smoking, alcohol use, BMI, and recorded comorbidities) together with the biomarkers. We additionally performed sensitivity analyses in the subset with more complete radiological data (including aneurysm size, location category, and shape) to assess the robustness of biomarker associations.

## 3 Results

### 3.1 General characteristics of the patient cohort

Of the 80 patients who underwent endovascular embolization, 27 had RA and 53 had UA. Baseline demographic and lifestyle characteristics available in this dataset (sex, age, BMI, smoking, and drinking) did not differ significantly between groups (Table 1). While several established rupture predictors (e.g., aneurysm size, location, and hypertension) were unavailable, we included all accessible baseline variables (age, sex, BMI, smoking, and drinking) in extended multivariable models. The biomarker associations with rupture remained statistically significant after this adjustment, suggesting robustness of the results.

### 3.2 Differences in inflammatory and metabolic biomarkers pre- to post-treatment

Across all patients, several pro-inflammatory and oxidative stress biomarkers decreased 1 week after endovascular treatment, including PLR, Lp-PLA2, MHR, SII, HCY, MDA, IL-6, and TNF-α (all *p* < 0.05; Table 2). In contrast, the anti-inflammatory/antioxidant markers SOD and IL-10 increased after treatment (*p* < 0.05).

**Table 2:** The levels of PLR, Lp-PLA2, MHR, SII, HCY, MDA, IL-6, TNF-α, SOD, and IL-10 in IA patients before and after treatment (*x* ± *s*).

Index	Before treatment	After treatment	*t*	*P*
PLR	98.65 ± 13.26	93.12 ± 14.59	2.509	0.013
Lp-PLA2 (μg/L)	168.06 ± 25.61	95.68 ± 16.02	21.431	0.000
MHR	0.42 ± 0.16	0.31 ± 0.18	4.085	0.000
SII	797.48 ± 93.02	435.16 ± 82.05	26.127	0.000
HCY (μmol/L)	15.53 ± 3.15	14.03 ± 2.58	3.295	0.001
MDA (μmol/L)	7.75 ± 1.35	5.35 ± 1.13	12.193	0.000
SOD (kU/L)	73.02 ± 8.03	90.11 ± 9.02	12.658	0.000
IL-6 (pg/mL)	281.03 ± 19.82	192.31 ± 20.16	28.069	0.000
IL-10 (pg/mL)	14.68 ± 2.15	21.06 ± 3.35	14.336	0.000
TNF-α (pg/mL)	442.06 ± 19.15	343.15 ± 20.15	31.825	0.000

Values represent mean ± standard deviation. Paired *t*-tests were used to compare pre- and post-treatment levels of each biomarker. Significant reductions were observed in PLR, Lp-PLA2, MHR, SII, HCY, MDA, IL-6, and TNF-α, while SOD and IL-10 increased significantly after treatment (all *P* < 0.05).

### 3.3 Comparison of ruptured and unruptured patients

Biomarker levels were compared between ruptured and unruptured patients using preoperative values. Ruptured patients showed higher levels of PLR, Lp-PLA2, MHR, SII, HCY, MDA, IL-6, and TNF-α, and lower levels of SOD and IL-10 (all *p* < 0.05; Table 3), indicating a more pro-inflammatory and pro-oxidative profile in ruptured cases.

**Table 3:** Comparison of Lp-PLA2, MHR, SII, HCY, MDA, IL-6, TNF-α, SOD, and IL-10 levels in patients with IA in the unruptured and the ruptured group (*x* ± *s*).

Index	Unruptured	Ruptured	*t*	*P*
PLR	97.13 ± 8.45	102.25 ± 8.68	2.860	0.005
Lp-PLA2 (μg/L)	162.13 ± 26.03	179.67 ± 24.87	2.892	0.005
MHR	0.38 ± 0.13	0.50 ± 0.17	3.511	0.001
SII	756.32 ± 96.98	878.26 ± 89.67	5.451	0.000
HCY (μmol/L)	14.39 ± 3.06	17.77 ± 4.13	4.139	0.000
MDA (μmol/L)	7.10 ± 1.21	9.03 ± 1.46	6.285	0.000
SOD (kU/L)	75.04 ± 7.64	69.03 ± 8.44	3.221	0.002
IL-6 (pg/mL)	258.63 ± 19.02	324.99 ± 20.34	14.415	0.000
IL-10 (pg/mL)	15.98 ± 3.21	12.12 ± 2.65	5.379	0.000
TNF-α (pg/mL)	413.38 ± 19.15	498.35 ± 20.64	18.280	0.000

Values are presented as mean ± standard deviation. Comparisons between groups were conducted using independent-sample *t*-tests. Ruptured patients showed significantly higher levels of PLR, Lp-PLA2, MHR, SII, HCY, MDA, IL-6, and TNF-α, and lower levels of SOD and IL-10 (all *P* < 0.05), indicating a more pro-inflammatory and pro-oxidative state.

Because rupture status is a binary outcome, we used univariate logistic regression to quantify the association between each biomarker and rupture. PLR, Lp-PLA2, MHR, SII, and HCY were each significantly associated with rupture (all *p* < 0.05), consistent with the between-group comparisons (Table 3). Correlation between biomarkers and aneurysm rupture risk in Supplementary Figure S1.

### 3.4 Multivariate analysis using a logistic regression model

Multivariate logistic regression confirmed that PLR, Lp-PLA2, MHR, SII, and HCY were independently associated with rupture (all *p* < 0.05; Table 4). In preliminary modeling using the original (unscaled) MHR values, the estimated odds ratio was extremely large with a very wide confidence interval, suggesting model instability due to scaling, sparse-data bias, or influential observations. Therefore, MHR was rescaled and entered per 0.1-unit increase, which yielded a more interpretable effect size (Table 4). We also examined multicollinearity (all VIFs < 5) and influence diagnostics (standardized residuals and Cook’s distance) to assess model robustness.

**Table 4:** Multivariate logistic regression analysis of risk factors for rupture of small and medium IA.

Influence factor	*B*	SE	Wald χ^2^	*P*	OR	95%*CI*	
						Lower limit	Upper limit
PLR	0.158	0.066	5.705	0.017	1.171	1.029	1.334
Lp-PLA2	0.059	0.025	5.437	0.020	1.061	1.009	1.115
MHR (per 0.1 increase)	0.773	0.375	4.237	0.040	2.166	1.038	4.520
SII	0.031	0.009	11.104	0.001	1.032	1.013	1.051
HCY	0.523	0.191	7.508	0.006	1.686	1.160	2.451
Constants	−64.230	18.588	11.940	0.001	–	–	–

Regression model including PLR, Lp-PLA2, MHR (scaled per 0.1 increase), SII, and HCY. All five biomarkers were identified as independent factors associated with rupture in small and medium-sized IAs (all *P* < 0.05). OR, odds ratio; CI, confidence interval.

### 3.5 Predictive risk model of biomarkers for rupture risk

**Figure 1:**
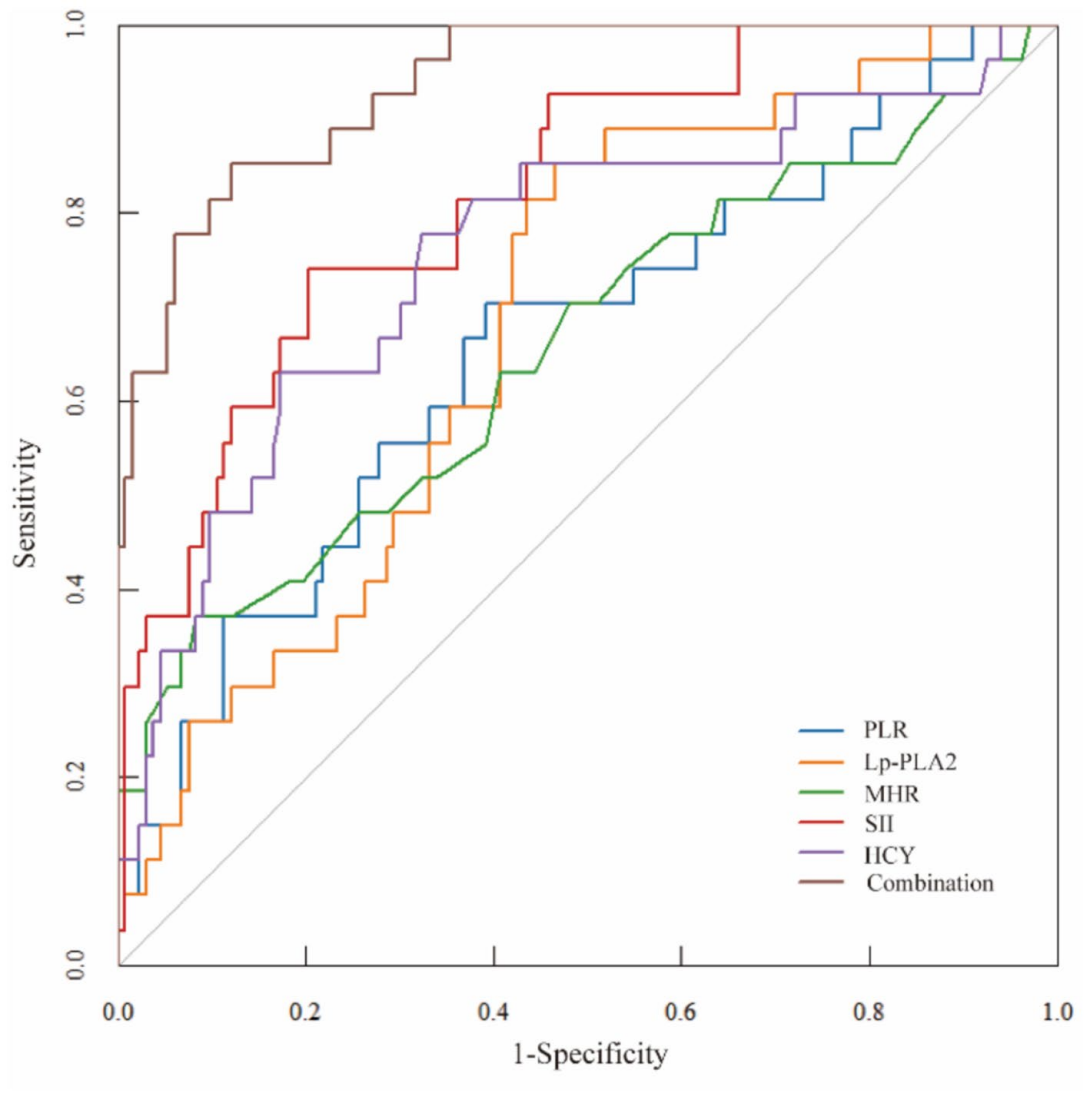
Biomarker values by rupture status. Plots showing PLR (ratio, unitless), Lp-PLA2 (μg/L), MHR (ratio), SII (10^9^/L), and HCY (μmol/L) stratified by rupture status (unruptured vs. ruptured).

**Figure 2:**
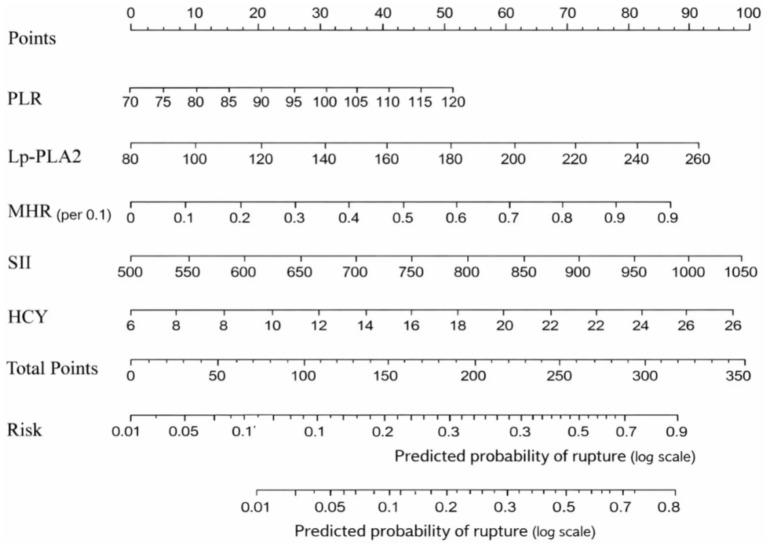
Nomogram for rupture risk prediction. A visual scoring tool integrating PLR (ratio, unitless), Lp-PLA2 (μg/L), MHR (ratio, per 0.1-unit increase), SII (10^9^/L; computed as platelet × neutrophil/lymphocyte with counts in 10^9^/L), and HCY (μmol/L). Each variable value is translated into points, summed, and mapped to predict individual rupture risk.

**Figure 3:**
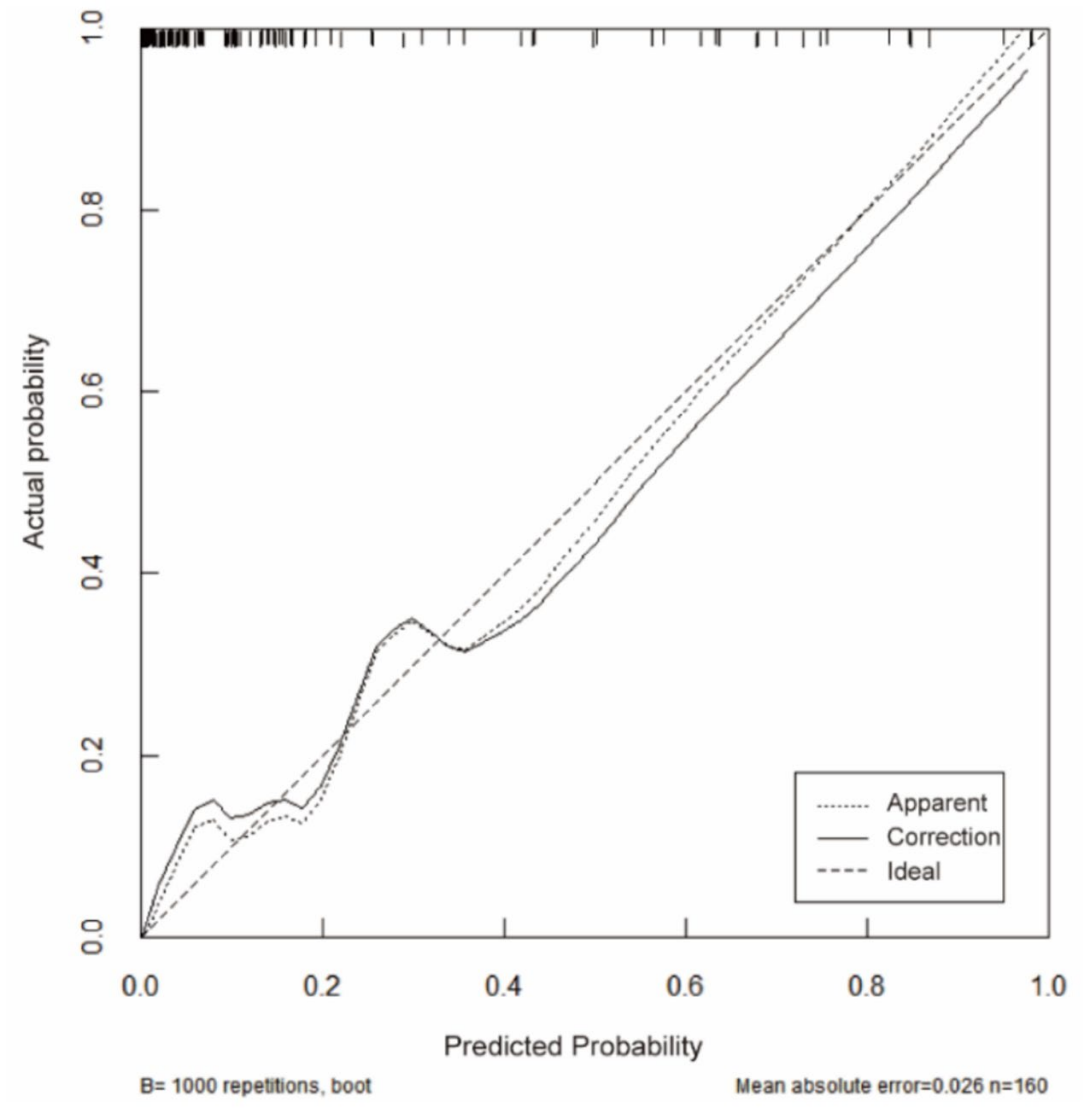
Calibration curve of the nomogram for rupture risk prediction. The plot compares predicted probabilities with observed outcomes. The dotted line represents the apparent performance, the solid line represents the bootstrap bias-corrected estimate (*B* = 1,000 resamples), and the dashed line indicates ideal calibration. Model calibration was also assessed using the Hosmer–Lemeshow goodness-of-fit test (*p* > 0.05 indicates no evidence of lack of fit).

**Figure 4:**
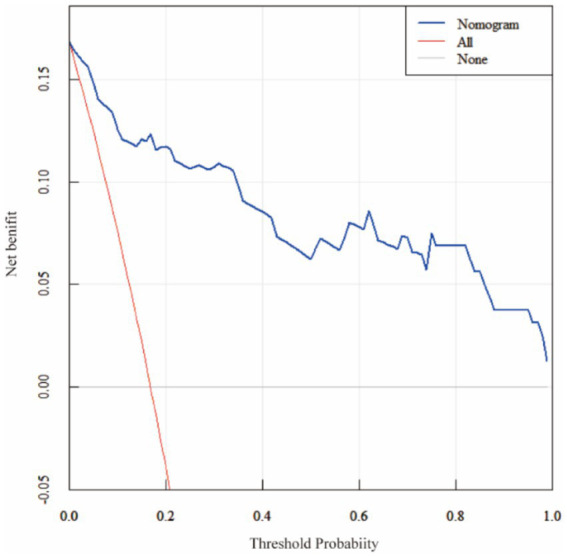
Decision curve analysis (DCA) of the nomogram. Decision curve analysis comparing the nomogram with treat-all and treat-none strategies. The nomogram yields a higher net benefit across a range of clinically relevant threshold probabilities (0.05–0.80).

ROC analysis demonstrated that SII had the highest predictive value among the five individual biomarkers (AUC = 0.837). The combined biomarker model showed high apparent discrimination (AUC = 0.969; Table 5; Figure 1). The predictive model on nomograph showed excellent calibration (Figure 2). Given the limited sample size and the single-center retrospective design, we performed bootstrap internal validation (1,000 resamples) to quantify optimism in discrimination and calibration. Calibration was evaluated using a calibration curve and the Hosmer–Lemeshow goodness-of-fit test (Figures 3, 4; Supplementary Table S2). Although traditional morphological variables were not fully available, we constructed an extended model including available demographic and lifestyle factors (age, sex, BMI, smoking, drinking), which showed similar discrimination performance compared to the biomarker-only model (Supplementary Table S3), supporting the stability of the biomarker signal.

**Table 5:** Analysis of predictive efficacy of PLR, Lp-PLA2, MHR, SII, and HCY in rupture of small and medium IA.

Index	Sensitivity (%)	Specificity (%)	Youden index	Cutoff value	AUC area	95%CI
PLR	81.5	52.8	0.343	97.505	0.676	0.555–0.797
Lp-PLA2	44.4	84.9	0.293	187.330	0.683	0.561–0.804
MHR	66.7	73.6	0.403	0.435	0.719	0.590–0.847
SII	92.6	62.3	0.549	777.325	0.837	0.750–0.924
HCY	66.7	84.9	0.516	17.115	0.753	0.632–0.874
Combination	96.3	88.7	0.850	–	0.969	0.937–1.000

Receiver operating characteristic (ROC) analysis showing sensitivity, specificity, Youden index, cutoff value, and AUC for each biomarker alone and the combined model. The combination of all five biomarkers achieved the highest apparent predictive accuracy (AUC = 0.969).

To explore the added value of biomarkers over basic demographic and lifestyle variables, we constructed an extended model including age, sex, BMI, smoking, and drinking. This traditional-factor-only model showed modest discrimination (AUC = 0.656, 95% CI: 0.512–0.781), while the biomarker-only model achieved AUC = 0.969. When both traditional and biomarker variables were combined, the AUC was 0.972 (95% CI: 0.942–1.000), suggesting that the five biomarkers provide substantial incremental predictive value over accessible clinical factors (Supplementary Figure S2).

### 3.6 Independent validation of the biomarker model

The combined biomarker model was applied to the independent same-center validation cohort of 50 patients (17 ruptured, 33 unruptured). The nomogram showed good discriminatory performance with an AUC of 0.901 (95% CI: 0.825–0.978), and acceptable calibration (Hosmer–Lemeshow *p* = 0.407). The ROC curve for the validation cohort is shown in Supplementary Figure S3. This finding suggests that the model maintains performance in an independent sample from the same hospital setting; however, validation across other centers and populations is still required to establish broader generalizability.

### 3.7 Sensitivity analysis adjusting for traditional risk factors

In a subset of 58 patients with available morphological data, we repeated the multivariable analysis including aneurysm size (>7 mm), location (posterior vs. anterior circulation), and shape (irregular/multilobulated). The associations between key biomarkers (e.g., MHR, SIRI) and rupture status remained statistically significant after adjusting for these variables (all *p* < 0.05), suggesting that the biomarker model retained predictive value beyond traditional morphological predictors (Table 6).

**Table 6:** Multivariable logistic regression analysis of rupture risk in the subgroup with available morphological data (*n* = 58).

Variable	Adjusted OR	95% CI (lower)	95% CI (upper)	*P* value
Biomarkers				
PLR (per 10 units)	1.24	1.04	1.48	0.018
Lp-PLA2 (per 10 μg/L)	1.08	1.01	1.16	0.032
MHR (per 0.1 unit)	2.35	1.12	4.93	0.024
SII (per 100 units)	1.15	1.06	1.25	0.001
HCY (per 1 μmol/L)	1.18	1.05	1.33	0.006
Traditional factors				
Aneurysm size (>7 mm)	2.89	1.12	7.45	0.028
Location (posterior circulation)	2.41	1.03	5.63	0.043
Shape (irregular/multilobulated)	1.96	0.87	4.41	0.104

## 4 Discussion

Intracranial aneurysms represent pathological dilatations of cerebral arterial walls, arising from complex interactions among hemodynamic stress, vascular remodeling, and underlying biological imbalances that compromise structural integrity (15, 16). Epidemiological data indicate that unruptured intracranial aneurysms occur in approximately 2.3–3.2% of the general population, most of which are not inherited (17). Although many of these lesions remain stable throughout life, aneurysm rupture carries devastating consequences, with mortality reported as high as 40–65% following recurrent hemorrhage (18). Thus, accurately stratifying rupture risk in patients with unruptured aneurysms is essential to guide clinical management and improve outcomes.

Our study systematically evaluated the combined predictive ability of five circulating biomarkers (PLR, Lp-PLA2, MHR, SII, and HCY) for rupture status among small and medium intracranial aneurysms (≤ 15 mm). Earlier studies have largely focused on individual markers or morphological risk scores. In this cohort, a biomarker-only nomogram showed high apparent discrimination (AUC = 0.969). However, because key clinical and morphological predictors were not available for inclusion, we cannot confirm whether the biomarker panel provides incremental predictive value beyond traditional risk factors. Thus, the current model should be interpreted as complementary to traditional risk stratification. Although key morphological predictors (e.g., size, location, shape) were unavailable in this dataset, our extended analyses showed that the biomarker panel provided substantial predictive discrimination beyond basic demographic and lifestyle factors. This supports the potential incremental value of incorporating inflammatory and metabolic biomarkers into rupture risk models. Future studies should integrate both morphological and biological features to more fully capture rupture risk.

Our findings are supported by established pathophysiological mechanisms: (1) An elevated PLR reflects a shift toward a pro-thrombotic state driven by platelets, coupled with diminished lymphocyte-mediated immunoregulation—a profile that may promote vascular inflammation and accelerate wall degeneration (19). (2) Lp-PLA2 directly contributes to the oxidative modification of low-density lipoprotein and hydrolyzes oxidized phospholipids, processes that can destabilize plaque and erode vascular integrity (20, 21). (3) A high MHR signals an increased monocyte burden—central to inflammatory lipid metabolism—alongside reduced anti-inflammatory protection from HDL. This milieu favors macrophage infiltration and matrix metalloproteinase (MMP) release, which degrade the extracellular matrix of the aneurysm wall (22, 23). (4) SII integrates platelet, neutrophil, and lymphocyte counts into a composite measure of systemic inflammation. Elevated SII marks a pro-thrombotic and pro-inflammatory environment that may facilitate wall-remodeling processes culminating in rupture. (5) Increased HCY levels induce oxidative stress, endothelial dysfunction, and vascular stiffening, all of which are recognized contributors to aneurysm pathogenesis (24, 25).

Our observations further substantiate the interplay between inflammation and oxidative stress in aneurysm rupture. Ruptured cases exhibited elevated levels of IL-6, TNF-α, and MDA, alongside reduced IL-10 and SOD—a pattern consistent with prior reports indicating that oxidative stress markers rise before aneurysm rupture (26, 27). The post-treatment decline in pro-inflammatory and pro-oxidative markers, together with an increase in anti-inflammatory and antioxidant mediators, suggests that endovascular embolization may confer biological benefits beyond mere mechanical isolation of the aneurysm sac, possibly through stabilization of the peri-aneurysmal vascular microenvironment (28–30).

Multivariable logistic regression confirmed that each biomarker independently predicted rupture risk. While SII demonstrated the highest discriminatory ability as a single predictor (AUC = 0.837), the combination of all five markers yielded the strongest overall performance and offered greater clinical utility for risk stratification. The resulting nomogram showed excellent calibration and provided meaningful net benefit across a range of decision thresholds. These findings support the potential of a multi-biomarker panel as a valuable adjunct to existing morphology-based scoring systems in clinical decision-making.

Several limitations should be acknowledged. First, this was a retrospective single-center study based exclusively on patients who underwent endovascular embolization. Ruptured cases were restricted to those with Hunt–Hess grades I–III at admission; consequently, the sample may not represent aneurysms managed conservatively, those treated with surgical clipping, or cases with more severe hemorrhagic presentations (Hunt–Hess grades IV–V). This introduces a potential selection bias that limits the generalizability of our findings to broader clinical populations. Moreover, the retrospective design precludes the establishment of causal relationships between the biomarkers and aneurysm rupture; the observed associations may reflect confounding by unmeasured factors or reverse causation. Therefore, the results should be interpreted as hypothesis-generating rather than definitive evidence of a predictive link. Second, the number of rupture events was small (*n* = 27), which increases the risk of model instability and optimistic discrimination; although we used bootstrap internal validation, the very high AUC should be interpreted cautiously and the model should be considered exploratory. Third, comprehensive adjustment for established rupture predictors was not feasible because several traditional clinical variables (e.g., detailed blood pressure measures, diabetes, lipid profile, medication use) and detailed radiological features (e.g., aspect ratio and quantitative irregularity/daughter sac features) were incompletely recorded. While we adjusted for available demographic and lifestyle factors and performed sensitivity analyses in a subset with more complete morphology (including size, location category, and shape), residual confounding may remain and biomarker effect estimates may be inflated. Fourth, the validation cohort was drawn from the same center; while it provides independent sample testing, true external validation in other institutions is lacking. Finally, we did not have longitudinal follow-up to evaluate temporal stability of biomarkers. Future multicenter prospective studies integrating comprehensive clinical, morphological, and biomarker information with external validation are warranted.

Future studies should focus on validating these findings through larger, multi-center prospective studies with longitudinal comparisons of biomarker profiles, and additional biological markers such as radiomics and hemodynamic modeling of risk. Such studies would allow us to verify either cause or predictive relationships to improve the clinical utility of combination multi-biomarker risk stratification tools.

## 5 Conclusion

PLR, Lp-PLA2, MHR, SII, and HCY were associated with rupture status in small to medium-sized intracranial aneurysms in this single-center retrospective cohort. A combined biomarker nomogram showed strong apparent discrimination; however, due to the limited number of rupture events and incomplete adjustment for established clinical and radiological predictors, the model may be subject to overfitting and residual confounding. While the biomarkers demonstrated robust associations after adjustment for available demographic and lifestyle factors, independent same-center validation showed preserved discrimination, but true external validation and prospective multicenter confirmation incorporating comprehensive traditional rupture predictors are required before routine clinical application.

## Data Availability

The original contributions presented in the study are included in the article/Supplementary material, further inquiries can be directed to the corresponding author.
