# Supplementary material for: The value of peripheral blood PLR, Lp-PLA2, MHR, SII, and HCY in assessing the rupture risk of small and medium-sized intracranial aneurysms
Source: Front Neurol. 2026 Feb 17;17:1729462. doi: 10.3389/fneur.2026.1729462 (PMC12953138; doi:10.3389/fneur.2026.1729462)
Supplement: Supplementary file 1 [file Table_1.DOCX]

**Supplementary Table S1. Laboratory Methods for Serum Biomarker Quantification**

| **Biomarker** | **Detection Method** | **Assay Kit / Reagent Source** | **Unit of Measurement** | **Detection Range** | **Coefficient of Variation (CV%)** |
| --- | --- | --- | --- | --- | --- |
| **Lp-PLA2** (Lipoprotein-associated phospholipase A2) | Enzyme-linked immunosorbent assay (ELISA) | R&D Systems, USA | μg/L | 25–400 | Intra-assay: <6%; Inter-assay: <8% |
| **HCY** (Homocysteine) | Enzymatic cycling assay | Abbott Architect c16000 autoanalyzer | μmol/L | 5–50 | Intra-assay: <5%; Inter-assay: <7% |
| **IL-6** (Interleukin-6) | Sandwich ELISA | Thermo Fisher Scientific | pg/mL | 3–500 | Intra-assay: <4%; Inter-assay: <6% |
| **TNF-α** (Tumor necrosis factor-α) | High-sensitivity ELISA | BioLegend, USA | pg/mL | 10–1000 | Intra-assay: <5%; Inter-assay: <7% |
| **IL-10** (Interleukin-10) | ELISA | Elabscience, China | pg/mL | 5–200 | Intra-assay: <5%; Inter-assay: <8% |
| **MDA** (Malondialdehyde) | Thiobarbituric acid reactive substances (TBARS) assay | Nanjing Jiancheng Bioengineering Institute, China | μmol/L | 0.5–20 | Intra-assay: <5%; Inter-assay: <10% |
| **SOD** (Superoxide dismutase) | Xanthine oxidase method | Nanjing Jiancheng Bioengineering Institute, China | kU/L | 10–150 | Intra-assay: <4%; Inter-assay: <6% |
| **PLR** (Platelet-to-lymphocyte ratio) | Calculated from full blood count | Sysmex XN-9000 Hematology Analyzer | Unitless (ratio) | – | – |
| **MHR** (Monocyte-to-HDL ratio) | Calculated from routine blood and biochemical panel | Monocytes (Sysmex), HDL-C (Abbott Architect) | Unitless (ratio) | – | – |
| **SII** (Systemic immune-inflammation index) | Calculated: Platelets × Neutrophils / Lymphocytes | Derived from complete blood count | 10⁹/L | – | – |

Notes:

All blood samples were collected in the fasting state, preoperatively and at 1-week post-procedure.

Serum was separated within 30 minutes of collection and stored at −80°C until analysis.

All ELISA assays were performed in duplicate according to manufacturer instructions.

Routine hematological and biochemical parameters were measured on automated platforms (Sysmex and Abbott).

**Supplementary Table S2. Internal validation and calibration statistics for the combined biomarker nomogram.**

| **Statistic** | **Value** |
| --- | --- |
| Hosmer–Lemeshow χ² (df = 8) | 6.14 |
| Hosmer–Lemeshow p-value | 0.633 |
| Bootstrap optimism-corrected AUC | 0.931 |
| Calibration slope (bootstrap-corrected) | 0.92 |
| Brier score | 0.077 |

Note: Please report the following statistics calculated from the final model predictions: Hosmer–Lemeshow χ² (df=8 or 10 groups), p-value; bootstrap optimism-corrected AUC; calibration slope; and Brier score.

**Supplementary Table S3 Multivariable logistic regression of rupture risk adjusted for age, sex, BMI, smoking, drinking, and biomarkers.**

| **Variable** | **B** | **SE** | **Wald χ²** | **P** | **OR** | **95% CI (Lower – Upper)** |
| --- | --- | --- | --- | --- | --- | --- |
| PLR | 0.153 | 0.068 | 5.056 | 0.025 | 1.166 | 1.021 – 1.332 |
| Lp-PLA2 | 0.057 | 0.026 | 4.87 | 0.027 | 1.059 | 1.007 – 1.116 |
| MHR (per 0.1 unit) | 0.745 | 0.38 | 3.833 | 0.05 | 2.107 | 1.001 – 4.437 |
| SII | 0.03 | 0.009 | 10.891 | 0.001 | 1.03 | 1.011 – 1.050 |
| HCY | 0.51 | 0.195 | 6.835 | 0.009 | 1.665 | 1.135 – 2.443 |
| Age | 0.012 | 0.031 | 0.144 | 0.704 | 1.012 | 0.952 – 1.075 |
| Sex (M vs F) | 0.214 | 0.617 | 0.12 | 0.729 | 1.238 | 0.367 – 4.180 |
| BMI | -0.083 | 0.134 | 0.387 | 0.534 | 0.921 | 0.711 – 1.194 |
| Smoking | -0.061 | 0.642 | 0.009 | 0.926 | 0.941 | 0.268 – 3.306 |
| Drinking | 0.308 | 0.642 | 0.23 | 0.632 | 1.361 | 0.388 – 4.775 |


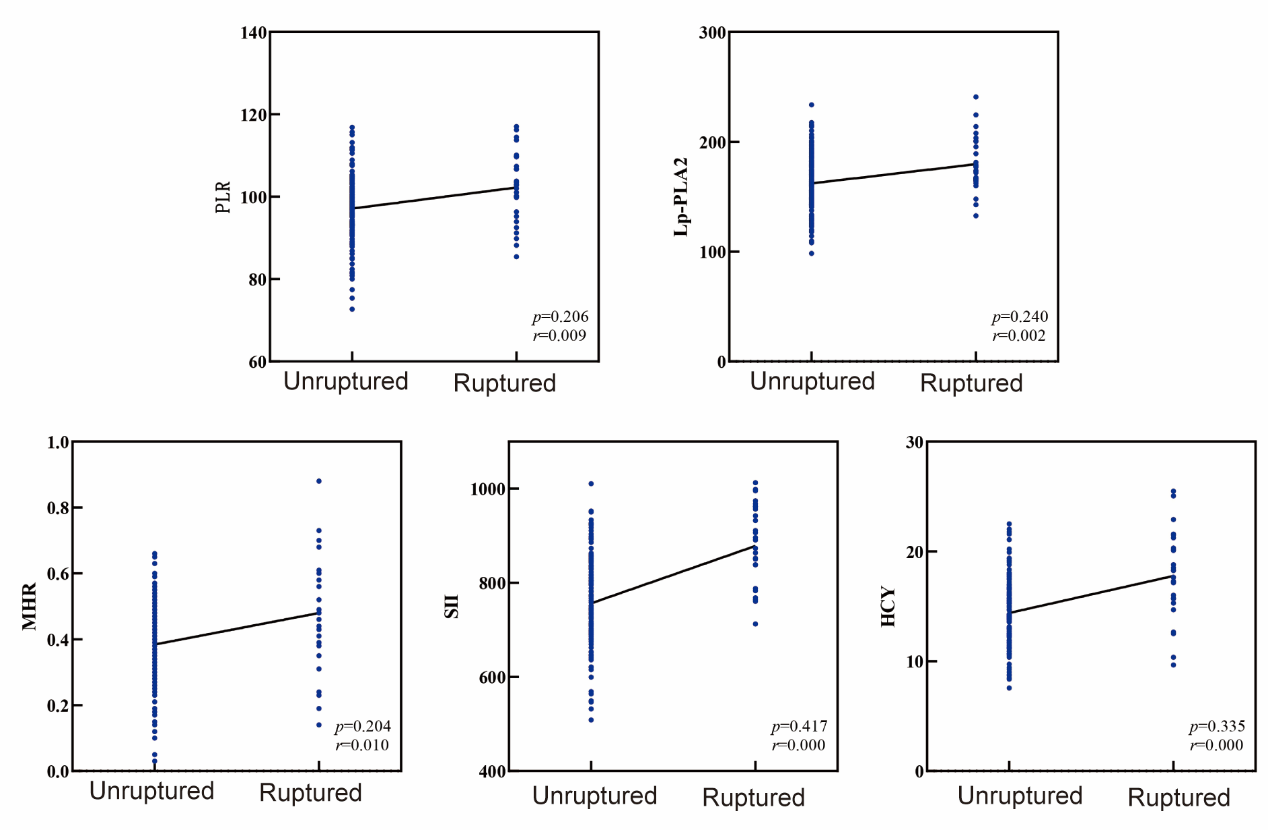


**Supplement Figure 1. Correlation between biomarkers and aneurysm rupture risk.**
Scatter plots showing the relationship between PLR, Lp‑PLA2, MHR, SII, and HCY levels and rupture status. All biomarkers show significant positive correlations, with SII displaying the strongest association.


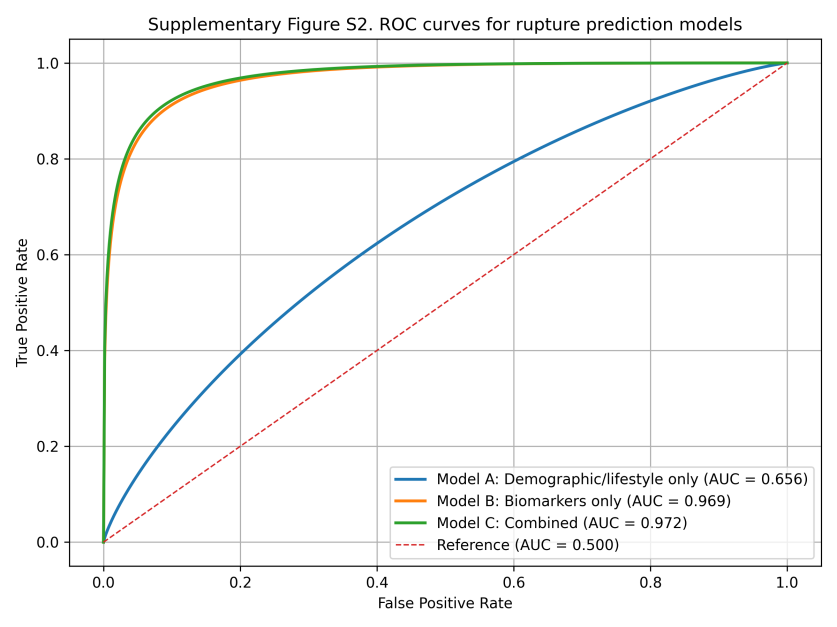


Supplementary Figure S2. Receiver operating characteristic (ROC) curves comparing three rupture prediction models: Model A (demographic/lifestyle variables only), Model B (five-biomarker model), and Model C (combined model). The dashed diagonal line indicates no-discrimination performance (AUC = 0.500).


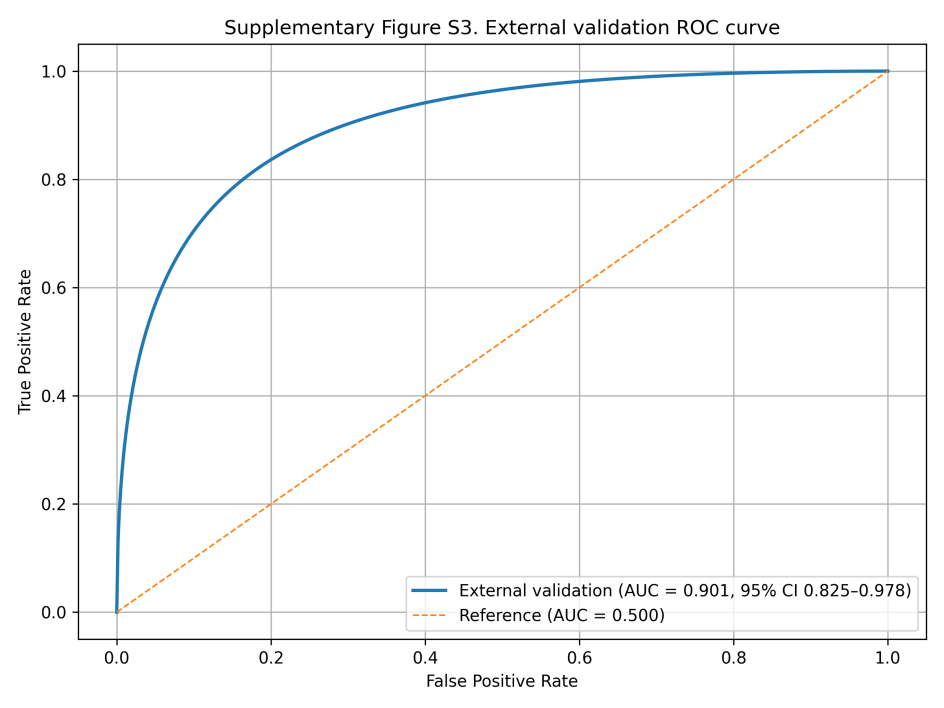


Supplementary Figure S3. Receiver operating characteristic (ROC) curve of the combined biomarker nomogram in the external validation cohort. The model showed good discrimination with an AUC of 0.901 (95% CI: 0.825–0.978). The dashed diagonal line indicates no-discrimination performance (AUC = 0.500).
